# The clinical utility of cardiac myosin inhibitors for the management of hypertrophic cardiomyopathy: a scoping review

**DOI:** 10.1007/s10741-024-10476-w

**Published:** 2024-12-17

**Authors:** Leen Othman, Lida Koskina, Nicholas Huerta, Shiavax J. Rao

**Affiliations:** 1 Department of Medicine, MedStar Union Memorial Hospital, Baltimore, MD USA; 2 Division of Cardiovascular Medicine, University of Virginia, 1215 Lee St Box 800158, Charlottesville, VA 22908 USA

**Keywords:** Cardiac myosin inhibitor, Hypertrophic cardiomyopathy, Hypertrophic obstructive cardiomyopathy, Aficamten, Mavacamten

## Abstract

Hypertrophic cardiomyopathy (HCM) is an inherited condition characterized by left ventricular, non-dilated hypertrophy in the absence of another secondary underlying cause. There has been an ongoing increase in the diagnosis of HCM over the past couple of decades, prompting further work in the area of pharmacological and interventional therapies. This scoping review aimed to summarize the traditional therapeutic options for HCM and to explore emerging research on novel cardiac myosin inhibitors (CMIs) as a new option for pharmacologic management of HCM. A PRISMA search strategy was carried out to identify the pertinent literature on mavacamten and aficamten—two novel CMIs. Seventeen studies were included. Based on the results of the studies included in this review, cardiac myosin inhibitors have been proven to be a safe and efficacious second-line option for the management of HCM. In the foreseeable future, based on results of ongoing studies investigating patient outcomes and side-effect profile, CMIs may potentially play a larger role as part of standard treatment of HCM.

## Introduction

What was once believed to be a rare inherited disease, hypertrophic cardiomyopathy (HCM) has been found to have a much higher worldwide distribution with an estimated prevalence of 1:200–1:500 [1]. Recent data have shown a significant shift in the epidemiological perception of HCM, with greater incidence in older patients, particularly with sporadic disease, genotype-negative status, and mild phenotypes [2]. There has been a significant increase in the diagnosis of HCM in patients over 60 years of age in United States (US) and non-US populations, from an estimated 9.2% prior to 2000 to an estimated 31.8% after 2010 [3]. Sex-specific differences may also exist for prognosis of HCM. A recent meta-analysis found that females have higher risk of HCM-related events and death [4]. This is in contrast to an older study analyzing US trends in HCM-related mortality using the CDC WONDER database which revealed that males had a twofold higher incidence of age-adjusted mortality rate than females [2]. It was also believed that in order for females to reach the diagnostic threshold of 15 mm wall thickness, they required a more severe phenotype and were diagnosed at later stages, thus undermining the true prevalence of the disease [3]. Using the same absolute cutoff for both sexes, as opposed to lower absolute values or indexed measurements, may explain the worse clinical outcomes in females. This is because LV thickness is associated with poorer survival at a lower threshold in females when compared to males [5]. Furthermore, non-Hispanic Black patients with HCM had the highest age adjusted mortality ratio in comparison to all races.

HCM can result from a mutation in one of the genes encoding cardiac sarcomere proteins, Z-disc proteins, or calcium controlling proteins [6]. Over 2000 mutations in 20 different genes have been identified in patients with HCM. The most common genes identified in HCM are B-myosin heavy chain (MYH7) and the myosin binding protein C (MYBPC3). Histopathological alterations occurring in HCM include hypertrophy, myofibril disruption, and increased interstitial fibrosis. These alterations can lead to systolic dysfunction, diastolic dysfunction, arrhythmias, and ischemia [6]. The hypertrophy can involve any area, but the interventricular septum is the most commonly affected. There have been two types of myocardial fibrosis described in HCM: replacement type and diffuse interstitial fibrosis [7]. Replacement fibrosis has been considered a culprit in end-stage (ES) HCM (defined as EF < 50%) because the fibrosis is present in all explanted hearts from patients with ES-HCM [7]. The considerable amount of fibrosis provides a reasonable explanation for the severely depressed LVEF in end-stage HCM patients. The mortality of ES-HCM is estimated to be around 11% per year [8], in contrast to 1% per year observed in classic HCM [9]. Genetic burden of sarcomeric and non-sarcomeric mutations may also be a risk factor for progression to ES-HCM [10–13]. Moreover, a stage termed adverse remodeling precedes ES-HCM [14]. Adverse remodeling occurs when the supranormal EF in classic HCM is decreased to the low-normal range of 50–60%. Medical management differs between the various stages of HCM so it is important to monitor clinical status, especially in patients on a calcium channel blocker or cardiac myosin inhibitor, as these medications will need to be discontinued in the event of LVEF reduction.

It was traditionally theorized that when septal hypertrophy is significant enough to cause stenosis of the left ventricular outflow tract (LVOT), the increased velocity of flow in the stenotic region leads to a regional decrease in pressure (Venturi effect) causing traction and systolic anterior motion (SAM) of the mitral valve leaflet during systole. This traction towards the interventricular septum subsequently results in a subaortic systolic gradient and LVOT obstruction (LVOT-O). However, this pathophysiology does not explain the persistence of SAM after septal reduction and newer theories are challenging this mechanism. Several authors suggest that drag forces, rather than the Venturi effect, are responsible for SAM of the mitral valve [15, 16]. This is supported by vector flow studies demonstrating anterior motion of the mitral valve actually begins during late diastolic inflow, creating a vortex which pushes the mitral valve anteriorly [17]. Regardless of the mechanism, it is important to recognize dynamic LVOT-O in clinical practice as the altered hemodynamics may lead to atypical responses to interventions (such as decreased cardiac output with inotropes). Additionally, increased LVOT gradient (LVOT-G) has been found to be an independent predictor of poor prognosis [18, 19].

The clinical diagnosis of HCM requires echocardiography or cardiac magnetic resonance imaging. For adults, a maximal end-diastolic left ventricular (LV) wall thickness $\ge$ 15 mm at any site or $\ge$ 13 mm in relatives of individuals with HCM or who are genotype positive is sufficient to make the diagnosis [20]. Obstructive HCM (oHCM) is diagnosed when the LVOT-G is ≥ 30 mmHg; otherwise, it is termed nonobstructive HCM (nHCM) when there is not a significant LVOT-G. One study found 37% of patients with HCM have LVOT-O at rest and an additional 33% develop obstruction with exercise [21]. Obstruction also seems to be provoked more frequently by exercise when compared to Valsalva [22]. Other evidence also suggests the prevalence of oHCM is around 56–70% of all individuals with HCM [22, 23]. Additionally, 90% of patients with HCM have ECG changes [24]. It is important to remember limitations of current diagnostic methods, such as inter- and intra-observer variability as well as atypical anatomy (i.e., hypertrophied crista supraventricularis) which may potentially lead to incorrect measurements. Additionally, as new data emerges, there may be more personalized diagnostic criteria in the future incorporating additional variables such as age and sex [25].

The clinical manifestations of HCM can vary widely and may present at any point in life. The majority of patients will have normal life expectancy without the need for major interventions. Recent data suggest that the estimated annual mortality of HCM range between 0.9 to 1.5% with a reduction of 0.5% per year following the introduction of new therapeutic interventions such as implantable cardioverter defibrillators (ICDs) and heart transplants [6]. HCM may also contribute to ischemic heart disease through several pathophysiologic mechanisms including increased oxygen demand secondary to increased muscle mass, insufficient coronary supply relative to the ventricular mass, and the increased diastolic pressures resulting in microvascular compression and reduced blood supply to the subendocardial region. Thickening of the coronary artery walls may be an additional contributing factor.

Historically, beta blockers (BB) and non-dihydropyridine calcium channel blockers (CCB) have been used as a first-line pharmacotherapy for the management of HCM. The goal of therapy is to decrease resting heart rate and decreasing oxygen demand while increasing the diastolic filling period. Disopyramide, an anti-arrhythmic agent, has also been used due to its negative inotropic effect which decreases the LVOT-G. In 2015, the INHERIT trial (Inhibition of the renin angiotensin system in hypertrophic cardiomyopathy and the effect on hypertrophy—a randomized intervention trial with losartan) failed to demonstrate reduction in LV mass with losartan as evidenced by MRI [26]. Subsequently, in 2021, the VANISH trial (valsartan for attenuating disease evolution in early sarcomeric HCM) met its primary composite outcome which included change in LV wall thickness, mass, and volumes, left atrial volume, tissue Doppler diastolic and systolic velocities, troponin, and BNP at 2 years. Other ongoing trials such as MERCUTIO and CIRRUS-HCM are investigating the effects of sarcomere modulators MYK-224 and EDG-7500, respectively.

When traditional treatment methods fail, invasive treatments are indicated in symptomatic patients with New York Hear Association (NYHA) functional classification III-IV who are on optimal medical therapy and have LVOT-G ≥ 50 mmHg. The invasive treatment option is septal reduction therapy (SRT), which typically includes surgical septal myectomy and alcohol septal ablation. Additionally, the septal scoring along midline endocardium (SESAME) procedure is a novel SRT currently under investigation [27]. Surgical septal myectomy has been considered the gold standard. It is established to reverse symptoms as well as restore normal LV filling pressures even in long-term studies with ≥ 10 years of follow-up [28]. Pacing therapy has also been investigated in patients with HCM using the rationale of reducing LV hyper-contractility through dyssynchronous activation of the septum, thus delaying septal thickening [29]. Pacing therapy additionally limits abnormal mitral valve motion and ventricular remodeling [6].

Most of the aforementioned therapies have been effective in reducing morbidity of the disease, but a significant unmet therapeutic need for obstructive and non-obstructive phenotypes still exists. Until recently, most therapies did not target the direct pathophysiology of HCM. However, in the recent years, tremendous developments have been made in transitioning such therapies from bench to bedside through the discovery of novel therapeutic agents. Novel cardiac myosin inhibitors (CMI), which target the core pathophysiological abnormality, have shown much promise over the past few years. Currently, two main cardiac myosin inhibitors are in the spotlight: mavacamten and aficamten. In this review, we summarize the recent clinical developments with respect to CMIs and their role in the management of HCM.

## Methods

**Fig. 1:**
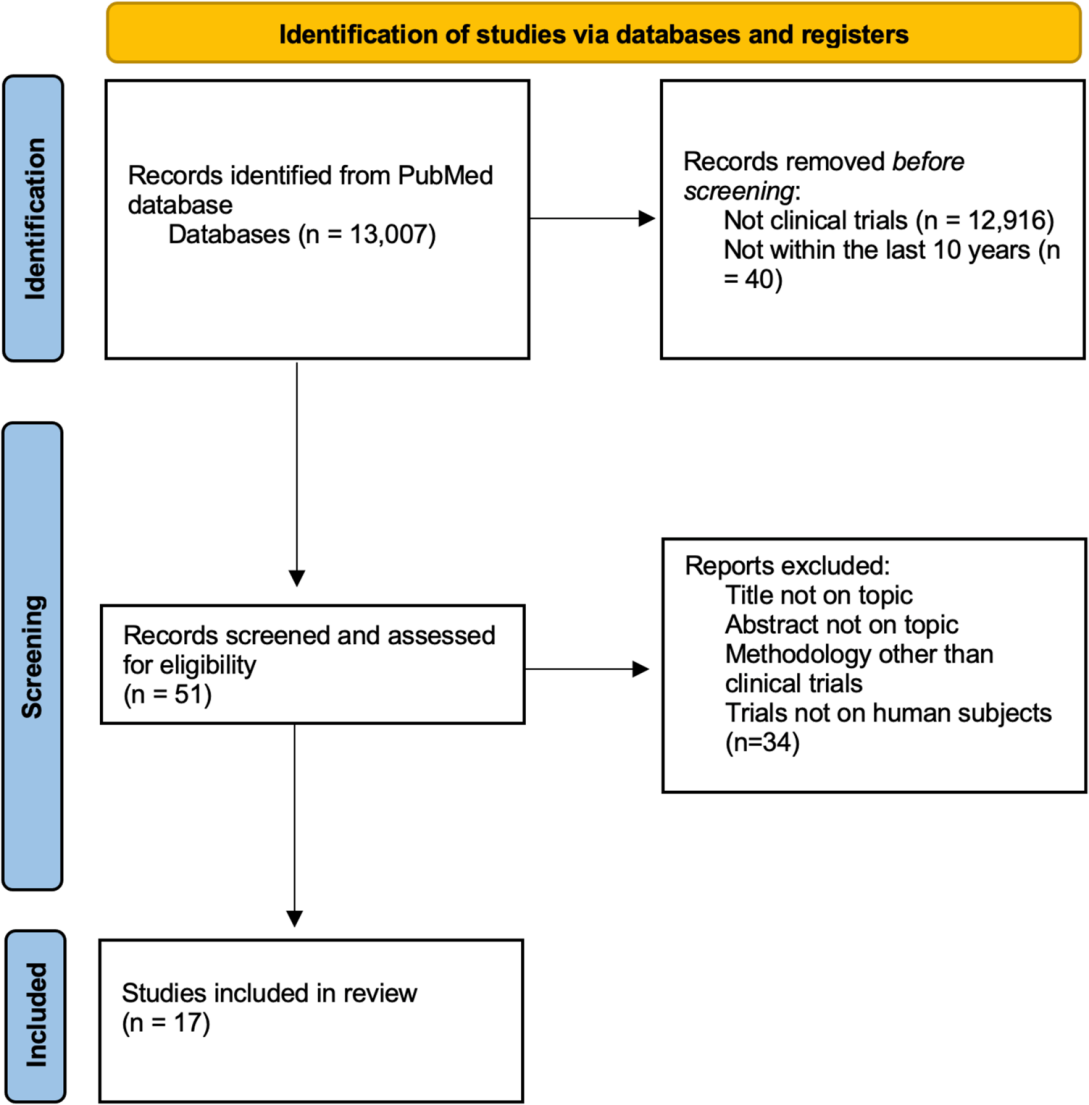
PRISMA flow diagram outlining identification, screening and inclusion of studies

To identify and retrieve the relevant articles, a comprehensive search of the PubMed database was conducted. A combination of Medical Subject Heading (MeSH) terms and search words related to the topic of interest were used. MeSH and search terms included “hypertrophic cardiomyopathy,” “mavacamten,” “aficamten,” and “sarcomere.” The filters “clinical trials” and “within the last 10 years” were applied. The initial search yielded 51 articles. Only English-language literature was included. Review articles and case reports were excluded. The titles and abstracts of the articles were screened further to determine eligibility and relevance to the topic and subtopics of interest. Thirty-four articles were excluded based on the title or abstract not being relevant to the topic of interest, the study design not being a clinical trial, or the study being conducted in non-human subjects. A total of 17 studies were included in the review. A flow diagram outlining the search strategy, screening, and data extraction is highlighted in Fig. 1. The studies investigating the utility of CMI in the management of HCM are summarized in Table 1.

**Table 1:** Studies investigating the utility of cardiac myosin inhibitors (mavacamten and aficamten) in the management of hypertrophic cardiomyopathy

Author	Year and Short Title	Purpose	Design	Endpoint(s)	Findings
Olivotto et al	2020 EXPLORER-HCM	Test efficacy of mavacamten	Randomized, double-blind, placebo-controlled, phase 3 trial	Composite of ≥ 1.5 mL/kg/min pVO_2_ increase and at least one NYHA class reduction or ≥ 3.0 mL/kg/min pVO_2_ increase without NYHA class worsening	Primary endpoint met with difference of 19.4%, CI = 8.7–30.1, *p* = 0.0005), post-exercise reduction in LVOT gradient with mavacamten with difference of − 36 mmHg, CI = − 43.2– − 28.1, *p* = < 0.0001
Ho et al	2020 MAVERICK-HCM	Test safety and tolerability of 16 week course of mavacamten at 200 ng/mL and 500 ng/mL	Multicenter, double-blind, placebo-controlled, dose ranging phase 2 trial	Safety and tolerability of 16-week course of mavacamten and exploratory endpoint included composite of ≥ 1.5 mL/kg/min pVO_2_ increase and at least one NYHA class reduction or ≥ 3.0 mL/kg/min pVO_2_ increase without NYHA class worsening	Met safety and tolerability endpoint, respective TEAE and SAE = 66 and 3 in 200 ng/mL group, 73 and 3 in 500 ng/mL group and 41 and 4 in placebo group, most common side effect was dizziness
Heitner et al	2019 PIONEER-HCM	Characterize effects of mavacamten on LVOT at doses of 10–20 mg/d without beta blockers (cohort A) and 2–5 mg/d with beta blockers (cohort B)	Open-label, nonrandomized, phase II trial	Primary endpoint was change in post-exercise LVOT at 12 weeks and secondary endpoints included changes in pVO2, LVEF, resting and Valsalva LVOT gradient, and numerical NRSDS	Cohort A mean LVOT change − 89.5 mmHg, CI = − 138– − 41, *p* = 0.008, Cohort B mean LVOT change = − 25 mmHg, CI = − 47– − 3, *p* = 0.020
Desai et al	2022 VALOR-HCM	Does mavacamten lead to enough improvement to forego SRT?	Double-blind, randomized clinical trial	Composite of the proportion of patients proceeding with SRT or remained guideline-eligible after 16 weeks	Difference in CMI group 58.6%, CI = 44–74%, *p* < 0.001. Secondary hierarchical testing favored mavacamten with mean difference post exercise LVOT gradient − 37.2 mmHg and ≥NYHA class
Maron et al	2023 REDWOOD-HCM	Evaluate safety and efficacy of aficamten in oHCM	Multicenter, randomized, double-blind, placebo controlled, phase II clinical trial	Safety, changes in gradient, LVEF, NYHA class, biomarkers	In aficamten group, mean resting gradient difference − 40 mmHg, *p* = < 0.05, mean EF difference −6, *p* < 0.05, non-significant changes in NYHA class, mean BNP reduction 62%, *p* = 0.0002
Wang et al	2024	Examine characteristics of patients with oHCM and HTN and response to mavacamten	Post hoc exploratory analysis of EXPLORER-HCM	Estimate treatment differences in those with HTN	Mavacamten group had improvements in all primary, secondary, and exploratory endpoints regardless of HTN status or mean SBP
Rader et al	2024 MAVA-LTE	Evaluate interim results of EXPLORER-LTE cohort from MAVA-LTE, which is mavacamten after withdrawal at the end of EXPLORER-HCM	Randomized, dose-blinded, extension study	Frequency and severity of TEAEs and SAEs with additional efficacy assessments	Total number of TEAEs = 895, total number of SAEs = 56, at 48 weeks mean changes of resting LVOT gradient = − 35.6, NT-proBNP = − 480 ng/L, and 67.5% improved by ≥ 1 NYHA class
Wheeler et al	2023	Determine the effects of background treatment on mavacamten outcomes	Subgroup analysis of EXPLORER-HCM and MAVA-LTE	N/A	75.3% on BB and 24.7% on CCB, 90.4% on BB had chronotropic incompetence, lower improvements in pVO2 with BB with mean difference 1.04 vs 2.69, Ve/VCO_2_ slope unaffected, BB had minimal impact on heart rate-independent measures but effected heart rate-dependent measures
Wheeler et al	2023	Investigate the effect of mavacamten on exercise physiology using CPET	Exploratory analysis of EXPLORER-HCM	VCO_2_, V_E_, peak Ve/VCO_2_, peak RER, peak circulatory power, ventilatory power, ventilatory threshold, peak MET’s, peak exercise time, PETCO_2_, VO_2_/workload	Significant improvements in almost all parameters
Desai et al	2022 VALOR-HCM-32-week	A study to evaluate mavacamten in adults with symptomatic oHCM who are eligible for SRT (VALOR-HCM)	Multicenter double-blind randomized placebo-controlled phase III trial	Proportion of patients proceeding with SRT or remaining guideline eligible at 32 weeks	10.7% in original mavacamten group and 13.5% of cross-over group met SRT guideline criteria or elected to undergo SRT at 32 weeks
Cremer et al	2022	Assess whether mavacamten improved echocardiographic features of diastolic function	Exploratory sub-study of VALOR-HCM	Composite of diastolic function parameters from baseline to week 16	Mavacamten group showed 29.4% improvement in diastolic function vs 12.8% in placebo group (*p* = 0.05)
Masri et al	2024 PIONEER-OLE	Long-term safety and effectiveness trial of mavacamten in oHCM	Ongoing, prospective, single-arm, open-label, phase II trial	Safety endpoints included CV death, sudden death, CV hospitalization, HFrEF, VF, VT, syncope, seizures, stroke, QT interval. Efficacy endpoints included NYHA and KCCQ	Treatment-emergent adverse events were mostly mild-moderate, 50% with NYHA improvements from baseline and mean KCCQ change 17
Bertero et al	2024	Compare real-world oHCM patients to those enrolled in EXPLORER-HCM	Real world comparison study	Proportion of patients potentially eligible to mavacamten according to EXPLORER-HCM	Total of 48.9% of patients would have been eligible for enrollment, compared to EXPLORER-HCM the real-world population as older, had lower BMI, and higher rates of AF
Roehl et al	2024	Evaluate response to mavacamten in oHCM subgroup which only obstructs with exercise or amyl nitrite	Single center retrospective observational	N/A	No improvement in NYHA class, significant reduction in LVOT gradient (− 48), higher reduction in EF (− 10%)
Xie et al	2022	Assess effects of mavacamten on HRQoL in EXPLORER-HCM cohort	Secondary analysis of randomized, double-blind, placebo controlled trial	EQ-5D-5L, EQ-VAS	Significant 30 week improvement in EQ-5D-5L (adjusted difference 0.073) and EQ-VAS (adjusted difference 7.5)
Hedge et al	2021	Evaluate effects of mavacamten on measures of cardiac structure and function in EXPLORER-HCM cohort	Secondary analysis of randomized, double-blind, placebo controlled trial	Changes in key echocardiographic parameters over 30 weeks	Complete resolution of mitral valve systolic anterior motion (difference 46.8%, *p* = < 0.0001), improvements in LAVI (mean − 7.5 mL/m^2^) and lateral E/e’(difference − 3.8, *p* < 0.0001)
Spertus et al	2021	Assess the effect of mavacamten on health status in EXPLORER-HCM	Secondary analysis of randomized, double-blind, placebo controlled trial	KCCQ change at 30 weeks	Greater KCCQ change in mavacamten group (difference 9.1, *p* < 0.0001), NNT = 5

*Abbreviations: pVO2* peak oxygen consumption, *NYHA* New York Heart Association, *CI* confidence interval, *LVOT* left ventricular outflow tract, *TEAE* treatment emergent adverse events, *SAE* serious adverse event, *LVEF* left ventricular ejection fraction, *NRSDS* numerical rating scale dyspnea score, *SRT* septal reduction therapy, oHCM obstructive hypertrophic cardiomyopathy, *HTN* hypertension, *SBP* systolic blood pressure, *BB* beta blocker, *CCB* calcium channel blocker, *CPET* cardiopulmonary exercise testing, *RER* respiratory exchange ratio, *MET* metabolic equivalents, *PETCO*_*2*_ partial pressure of end-tidal carbon dioxide, *Ve* minute ventilation, *VF* ventricular fibrillation, *VT* ventricular tachycardia, *KCCQ* Kansas City cardiomyopathy questionnaire, *AF* atrial fibrillation, *HRQoL* health-related quality-of-life, *EQ-5D-5L* EuroQoL 5-dimension 5-level index score, EQ-VAS EuroQoL visual analog scale, *LAVI* left atrial volume index, *NNT* number needed to treat

## Traditional pharmacotherapy

### Beta blockers

Non-vasodilating beta blockers (BB) are currently given a class I recommendations based on American and European guidelines for symptomatic HCM with and without obstruction [20, 30]. A seminal paper by Braunwald and Cohen in 1967 showed that propranolol reduced angina and improved exercise tolerance in patient with HCM (at the time it was termed idiopathic hypertrophic subaortic stenosis) [31]. Since then, others have replicated these findings [32, 33]. Beta blockers may also reduce the chances of sudden cardiac death [34]. There are observational studies showing a possible mortality benefit of BB but there are is a paucity of evidence from randomized controlled trials [35, 36]. Patients with HCM suffer from symptoms akin to heart failure for reasons already discussed. Beta blockers likely lead to improvement in these symptoms through negative chronotropic effects leading to increased end diastolic volume, increased systolic volume, reduced LVOT-O, and reduced mitral regurgitation [37].

### Calcium channel blockers

Non-dihydropyridine calcium channel blockers (CCB) are currently recommended for symptomatic HCM with and without obstruction. In the 2024 American guidelines when EF is preserved with symptomatic oHCM, BB are the preferred initial pharmacotherapy, with CCB being recommended when BB are ineffective or contraindicated as a class I recommendation [20]. However for symptomatic nHCM, CCB and BB are interchangeable first-line agents as a class I recommendation. In the 2023 European guidelines, CCB can be considered first-line for asymptomatic patients with a resting or provocable LVOT-G > 50 mmHg as a class IIb recommendation. However, in symptomatic patients, it is recommended to start with BB and add CCB if additional symptom control is needed or BB are contraindicated, in the absence of systolic dysfunction [30]. Both verapamil and diltiazem seem to lead to similar symptomatic benefits when compared to BB [38, 39]. Mechanistically, CCB may also have the added benefit of improving the impaired calcium homeostasis which occurs in HCM by blocking L-type calcium channels [40]. If the EF becomes reduced, guideline-directed medical therapy (GDMT) should be introduced and CCBs should be discontinued [20]. Both beta blockers and CCBs share a range of widely known side effects including bradyarrhythmia, AV block, generalized fatigue, and sexual dysfunction, as well as GI disturbances. Most of these medications are, however, well tolerated with minimal dose adjustments [41].

### Antiarrhythmics

Disopyramide is a class Ia antiarrhythmic. It is currently given a class I recommendation in combination with an AV nodal blocking agent for patients with obstruction and persistent symptoms despite treatment with BB and/or CCB. One of the first studies in five patients by Pollick et al. demonstrated acute decreases in resting subaortic gradient and systolic anterior motion of the anterior mitral leaflet and improved exercise tolerance with chronic use [42]. Several studies have shown symptomatic benefit in this patient population [43–45]. However, disopyramide should be used with caution in patients at high risk for torsades de pointes, as it can prolong the QT, even at low doses [46]. It should also be used in combination with an AV blocking agent due to its positive dromotropic effects to avoid precipitating arrhythmias with rapid ventricular response [20].

Disopyramide exerts a positive dromotropic effect at the AV node, which is why it is important to use it in conjunction with an AV nodal blocking agent to avoid arrhythmias. It may also lead to QT prolongation.

Cibenzoline is also classified as a type Ia antiarrhythmic. This drug was synthesized in Japan and is primarily used in Japan and Korea. It is currently not approved by the US FDA and is not mentioned in the current American or European guidelines. Hamada et al. have published several studies showing dramatic improvement in the LV pressure gradient, improvements in diastolic function, reductions in complication rates, reductions in death, and even regression of LV hypertrophy associated with cibenzoline [47–49]. Cibenzoline exerts negative inotropic effects and also indirectly effects calcium homeostasis in a similar manner to other ion channel blockers. A phase I clinical trial (NCT04418297) for a stereoisomer known as CT-G20 (s-cibenzoline) was terminated in the year 2023 because termination criteria were met during dose escalation, but there is no publication conveying the ultimate results [50].

### Ion channel blockers

Ion channel blockers such as ranolazine are not mentioned in the new American guidelines. However, the current European guidelines recommend to consider ranolazine to alleviate symptoms in patients experiencing angina-like chest pain, even when LVOT-O or obstructive coronary artery disease is not present. Ranolazine may lead to symptomatic improvement through several mechanisms. First, it acts via inhibition of the late phase of inward sodium channels which leads to decreased intracellular calcium and indirect reductions in calcium through increased export via the Na-Ca exchanger [51]. These changes may influence contractility and oxygen consumption. It also promotes glucose oxidation while reducing lactic acid production, which may lead to improved metabolic function of cardiac myocytes. There is non-randomized study data to support its use [52, 53]. However, the randomized clinical trial RESTYLE-HCM failed to show any benefit on exercise performance, brain natriuretic peptide (BNP) levels, diastolic function, or quality of life [54]. Another late sodium channel blocker known as eleclazine was developed by Gilead. It was tested in a phase II trial known as Liberty-HCM, but this trial was terminated prematurely due to the drug’s failure in a different trial (TEMPO) and a discontinuation of the entire eleclazine development program [55].

## Novel pharmacotherapies—cardiac myosin inhibitors

### Mavacamten

**Fig. 2:**
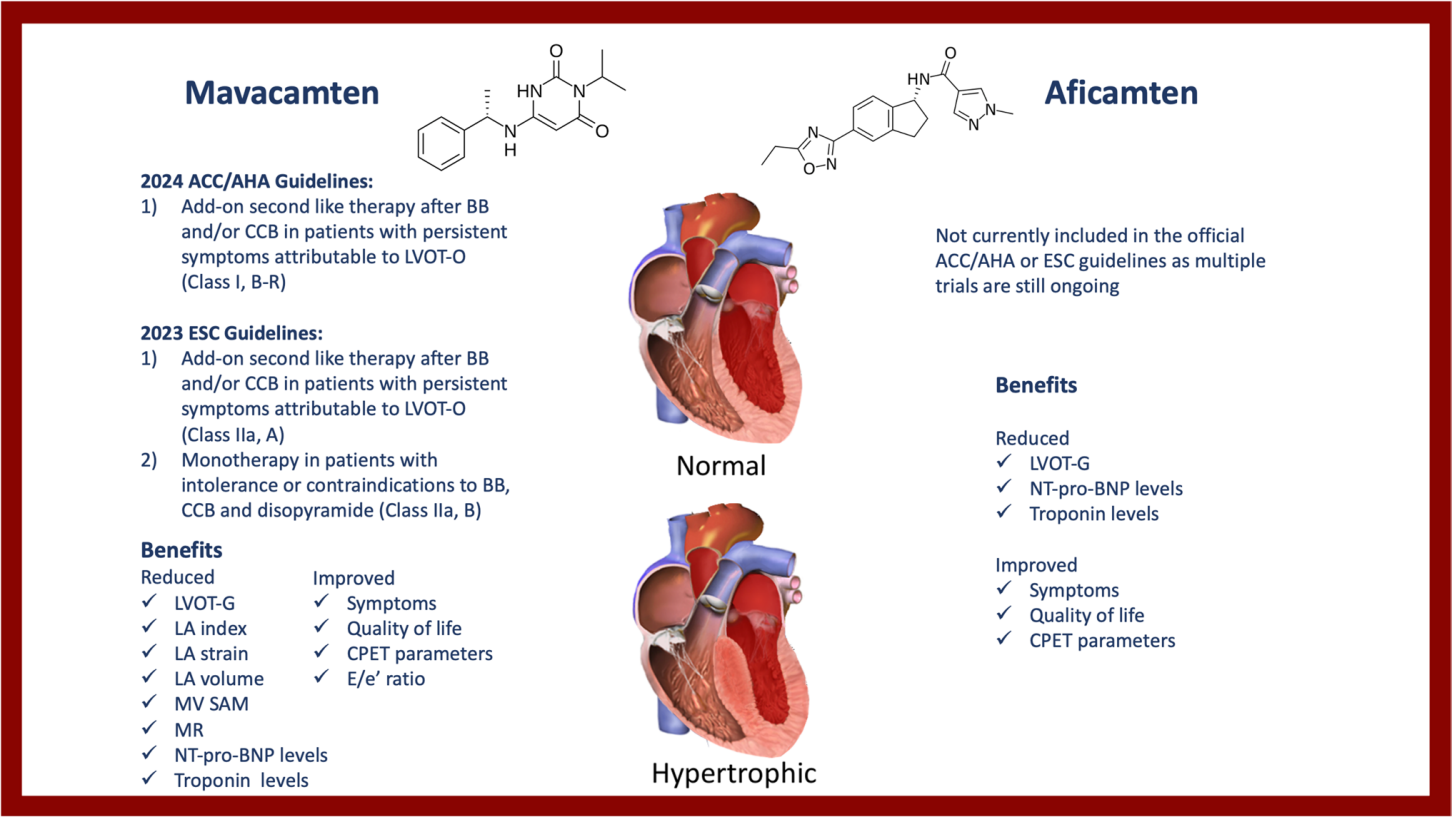
Central illustration. Overview of cardiac myosin inhibitors and their clinical utility in the management of hypertrophic cardiomyopathy. *Illustration Attribution: Npatchett at English Wikipedia, shared under the terms of the GNU Free Documentation License, Version 1.2 (https://www.gnu.org/licenses/old-licenses/fdl-1.2.en.html#SEC1).

Mavacamten is a selective allosteric reversible cardiac myosin inhibitor (Fig. 2). Specifically, this molecule binds to adenosine triphosphate (ATPase) in the cardiac myocytes and disables its attachment to the myosin heads. This results in an increased percentage of myosin found in a relaxed state which leads to a reduction in myocardial contractility. Furthermore, mavacamten can stabilize the relaxed state of the beta-cardiac myosin which further reduces myocardial contractility by interfering with actin-myosin coupling. Mavacamten is metabolized by cytochrome P450 family of enzymes, mainly CYP2C19 and CYP3A4. The half-life is between 6 and 23 days, depending on the action of the aforementioned enzymes [56].

Four landmark clinical trials have investigated the role of mavacamten in the treatment of HCM. The first such trial in 2019 was PIONEER-HCM, an open label, non-randomized, phase II clinical trial with a small cohort of 21 patients [57]. The primary endpoint was change in post-exercise LVOT-G after 12 weeks of therapy. The results were positive, showing a greater reduction in LVOT-G and maximal peak VO2 in patients treated with the higher dose (10–20 mg/day) as compared to patients treated with the lower dose (2–5 mg/day) plus beta-blocker. A year later, in 2020, MAVERICK-HCM was published. MAVERICK-HCM mainly looked at nonobstructive symptomatic HCM. This was a multicenter, randomized, double-blind, placebo-controlled, phase II clinical trial which recruited 59 patients [58]. Inclusion criteria included patients with NYHA functional class II/III, LVEF ⪰55% and NT-proBNP value of ⪰300 pg/mL. The participants in the trial were randomized in a 1:1:1 ratio to mavacamten at pharmacokinetic-adjusted-dose or placebo. The treatment was for 16 weeks followed by 8-week washout period. The starting dose was 5 mg daily and one dose titration occurred at week 6.

The primary endpoint was efficacy and safety of mavacamten. The study was underpowered to detect clinical benefit but the results were positive for a significant reduction in NT-pro-BNP and cardiac Troponin-I (cTnI) levels in the intervention group as compared to the placebo group. This offers a promising field where mavacamten can also provide significant value in the management of nonobstructive HCM [58].

Later that same year, EXPLORER-HCM was published. This was a multicenter, randomized, double-blind, placebo controlled, phase III clinical trial with a total of 251 patients [59]. The primary endpoints were improvement in peak oxygen uptake (pVO_2_) of ≥ 1.5 mL/kg/min and reduction of at least one NYHA class or improvement in exercise pVO_2_ of ≥ 3.0 mL/kg/min without NYHA class worsening. The results were revolutionizing, showing significant improvements in pVO_2_ and NYHA class in patients receiving mavacamten versus control. Secondary endpoints also showed greater reductions in post-exercise LVOT-G. The most exciting finding of this study was that 27% of patients in the intervention group achieved a complete response, defined as a reduction in all LVOT gradients to < 30 mmHg and attainment of NYHA class I symptoms after treatment, as opposed to only 1% achieving this response in the placebo group. Several subgroup analyses on the EXPLORER-HCM cohort have also been published. Xie et al. and Spertus et al. showed improved quality of life scores, Wang et al. showed improvements from mavacamten regardless of blood pressure status, Hegde et al. showed improved echocardiographic parameters such as left atrial volume index, and Wheeler et al. showed increased chronotropic incompetence and lower pVO2 improvements in those taking a BB or CCB and improvements in almost every parameter of exercise tolerance measured by cardiopulmonary exercise testing (CPET) [60–65]. Given the highly selective nature of trials, Bertero et al. also carried out a real-world comparison study and found that roughly half of the participants would have met eligibility criteria for EXPLORER-HCM [66].

Subsequently, the VALOR-HCM study, published in 2022, examined the efficacy and safety of mavacamten in 112 patients who met criteria for septal reduction therapy (SRT) or had been referred for the procedure [67]. VALOR-HCM was a randomized, double blinded, parallel-assignment, placebo-controlled phase III trial and the primary endpoint was the proportion of patients proceeding with SRT or remaining eligible for the procedure after 16 weeks of therapy. The results were again in favor of mavacamten treatment, showing that 18% of patients remained SRT eligible in the intervention group as compared to 77% of patients in the intervention group. Other secondary endpoints included a significant improvement in NYHA functional class and a significant reduction in resting and provoked LVOT-G in the mavacamten group as compared to the placebo group. Interestingly, in a subsequent analysis of the VALOR-HCM trial looking at changes in LA strain measures, mavacamten resulted in an improvement in the LA strain and LA volume index (LAVI) measurements at week 56. At baseline, the mean LA volume index measured was 41.3 ± 16.5 mL/m^2^, and the mean LA strain values were 11.8% ± 6.5% for conduit, − 8.7% ± 5.0% for contraction, and 20.5% ± 8.7% for reservoir. All of these values are noted to be worse than normal. Mavacamten demonstrated significant improvement in LAVI by − 5.6 ± 9.7 mL/m^2^ from baseline at week 56 (*p* < 0.001). A significant reduction in the absolute LA strain values was also demonstrated by 1.7 ± 6% for conduit, 1.2 ± 4.5% for contraction, and 2.8 ± 7.7% for reservoir. However, there was no significant improvement in LA strain values in patients with atrial fibrillation. This suggests favorable LA remodeling and improved function secondary to mavacamten in all patient groups except in patients with atrial fibrillation [68].

Another sub-analysis of the original VALOR-HCM cohort examined the long-term change in health status at the 56-week mark. The highest benefit of mavacamten therapy, as measured by the KCCQ-23 and KCCQ-OSS, was observed during the first 16 weeks of treatment and was sustained through week 56. In detail, patients in the mavacamten group had better health status measurements at week 16, with 65.5% of patients improving by > 5 on the KCCQ as opposed to 35.8% in the placebo group. Moreover, patients who were originally assigned to the placebo group had a dramatic improvement in their health status measurements after crossing over to the mavacamten group. Importantly, at 56 weeks, health status measurements were similar for both the early and delayed mavacamten initiation groups, suggesting that mavacamten has a fast and sustained benefit in quality of life measures [69].

Furthermore, mavacamten was shown to improve mitral regurgitation (MR) in the VALOR-HCM cohort. An exploratory substudy showed that MR improved in 48.1% of patients receiving the study drug as opposed to 12.2% of patients in the placebo group (*p* < 0.001). Moreover, an improvement in MR by 2 or more grades was found in 23.1% of patients in the mavacamten group when compared to 6.1% in the placebo group (*p* = 0.025). Mavacamten treatment and reduction in resting LVOT-G were found to be independent predictors of MR improvement in a multivariate Cox model. Improvement in systolic anterior motion (SAM) of the mitral valve was also observed more frequently in the mavacamten group as compared to the placebo group (53.7% vs 21.6%; *p* < 0.001) and this remained significant even when SAM improved by at least 2 grades (35.2% vs 11.8%; *p* = 0.007). However, it is important to note that these results are only hypothesis generating, given the small sample size and difficulty in quantifying MR volume in patients with HCM via echocardiography alone. Regardless, this is a promising observation which may be contributing to the efficacious effects of mavacamten [70].

Finally, Cremer et al. looked at the effect of mavacamten on left ventricular function, in 98 participants of the VALOR-HCM trial, both at baseline and at week 16. Of those treated with mavacamten, 29.4% had improvement in their diastolic function grade in comparison to 12.8% treated with placebo (*p* = 0.05). This was demonstrated through a decrease in the E/e’ ratio in patients treated with mavacamten compared to placebo (− 3.4 ± 5.3 vs 0.57 ± 3.5; *p* < 0.001). There was also a significant decrease in the indexed left atrial volumes in patients who received mavacamten compared with placebo (− 5.2 ± 7.8 vs − 0.51 ± 8.1; *p* = 0.005). The results remained significant even after adjustment for LVOT-G [71].

Primary results of the EMBARK-HFpEF trial were published in September 2024. This is a phase IIa, open label, one-arm trial investigating the effects of mavacamten in patients with HFpEF and LVEF > 60%. The primary endpoints included changes in levels of NT-pro-BNP, hs-Troponin T and I, NYHA class, and echocardiographic diastolic function parameters both at rest and during peak exercise. Safety endpoints were measured as a reduction of LVEF to < 30% or treatment-related adverse events. The patients were followed for 26 weeks. At the end of the follow-up period, NT-pro-BNP and troponin levels were significantly reduced. NYHA functional status improved in 41.7% of patients. One of the most important findings was the significant improvement in markers of LV diastolic function as measured by E/e’ ratio and LA volume. LV global longitudinal strain remained stable, whereas LVEF decreased only slightly during the treatment period. These changes were attributed to mavacamten given that most of these values returned to baseline after about 8 weeks of treatment discontinuation. Therefore, mavacamten seems to be a promising treatment strategy in patients with HCM and HFpEF [72].

Mavacamten has an overall favorable safety profile with only small percentages of mild and moderate adverse events and rare serious adverse events. In PIONEER-OLE (*n* = 13), only 1 out of 13 patients suffered an LVEF reduction to < 50%, with resolution after medication discontinuation, and no cardiovascular-related events, including hospitalization or death, were observed. Troponin elevation, atrial fibrillation, and QT prolongation were rare. Overall, there were six (38.5%) serious adverse events (SAE) but none was deemed to be related to the investigational drug. In the MAVA-LTE trial, the percentage of patients who suffered from cardiac failure was 3.5%, with all patients recovering their EF to > 50% after drug discontinuation. Other common adverse events included atrial fibrillation (9.1%), fatigue (10.4%), dizziness (10%), and hypertension (10%). The percentage of adverse events related to the investigational drug was 17%, of which 2.2% were serious (three patients with cardiac failure and two patients with reduced EF < 50%). Overall, 10 out of 201 had to permanently discontinue mavacamten. Death occurred in three patients, but the causes were considered unrelated to the investigational drug [59]. The evidence from the long-term extension cohorts, as described above, supports that mavacamten is generally safe and well-tolerated. However, heart failure is a significant potential side effect of mavacamten which necessitates close monitoring of patients on this treatment.

Mavacamten has been included in the newest 2024 AHA/ACC guidelines for the management of HCM [20]. The American guidelines do not specify dosage regimens when initiating this medication. The European guidelines mention that CMI can be initiated and titrated to a maximal tolerated dose [30]. Both guidelines recommend using mavacamten as a second-line therapy, in patients with symptomatic HCM secondary to LVOT-O in whom BB and/or CCB have failed or are contraindicated. The American guidelines give mavacamten a class I recommendation as opposed to the European guidelines which give mavacamten a class IIa recommendation. Furthermore, in the European guidelines, mavacamten is proposed as monotherapy in patients with intolerance or contraindications to the use of BB, CCB, and disopyramide as a class IIa, B recommendation. The American guidelines do not currently recommend CMI use as monotherapy. The medication is currently reserved only for adult patients and is available in 2.5 mg, 5 mg, 10 mg, and 15 mg capsules. Per the United States FDA, the recommended starting dosing is 5 mg daily, up-titrated over the span of at least 3 months to a maximum of 15 mg daily [60, 61]. However, the lowest effective dose should be used, and titration to the maximum of 15 mg daily is not necessary if symptoms are controlled and LVOT-O is < 20% with lower doses. Contraindications to the use of mavacamten mentioned by the FDA include concomitant use of moderate-to-strong CYP2C19/CYP3A4 inhibitors or inducers [61]. Additionally, the FDA does not recommend its initiation in patients with LVEF < 55% and therapy should be interrupted if LVEF decreases to < 50% [61]. However, mavacamten can be re-initiated at a lower starting dose of 2.5 mg, with close monitoring, in patients with recovery of LV systolic function. Per the FDA, caution should also be exercised when CMI is used with multiple other negative inotropic agents (disopyramide, ranolazine, combination of BB and CCB), although this is not an absolute contraindication to its use (FDA) [73].

### Aficamten

Aficamten is considered a next-generation, oral, small molecule inhibitor (Fig. 2). It works by decreasing the interaction between myosin heads and actin filaments, and therefore lowers the contractility force and decreases the LVOT-G. In terms of pharmacodynamics, aficamten exhibits a shorter half time compared to mavacamten, which is also suitable for once daily dosing. It reaches its steady state within 2 weeks [74]. It was also shown through in vivo studies to have minimal cytochrome P450 induction or inhibition and exhibits a wide therapeutic window [51]. Aficamten is one of the new myosin modulators which is specifically classified as a myosin inhibitor. Myosin exists in three main states that include active cycling, disordered relaxed, and super relaxed. Aficamten, similar to mavacamten, acts through stabilization of the energy-conserving super relaxed state, thus reducing LVOT-O and increasing cardiac filling pressures [74].

Aficamten (CK-274) was developed by cytokinetics as a selective small-molecule inhibitor of the myosin ATPase. In 2022, the first in-human phase I randomized, double-blinded, placebo-controlled trial examined the pharmacokinetic characteristics, safety, tolerability, and pharmacologically active ranges of the drug’s exposure and doses in healthy individuals. The study demonstrated that aficamten exhibited a half-life that is appropriate for once daily dosing and that the drug achieved steady state within 2 weeks. It also demonstrated a shallow exposure–response relationship and reversibility of the drug effect within 24 h of discontinuation with no significant drug-drug interactions [75].

In 2023, a phase II, multicenter, randomized double-blind, placebo controlled trial titled REDWOOD-HCM (Randomized Evaluation of Dosing with CK-3773274 in Obstructive Outflow Disease in HCM) was published [76]. This study aimed to assess the safety and efficacy of aficamten in treating oHCM on top of BB or CCB. The study spread out across 30 academic centers and involved 41 participants that were separated into two sequential cohorts and received overlapping aficamten doses. The patients were randomized in a 2:1 ratio to either receiving daily aficamten or placebo for a time period of up to 10 weeks. The participants in cohort 1 received 5–15 mg of aficamten while those in cohort 2 received 10–30 mg of the drug. During the 10-week study period and 2 weeks after the last dose, the participants were evaluated with echocardiography, laboratory diagnostics, and clinically. The primary endpoint of the trial was to evaluate the safety and efficacy of different aficamten doses by the occurrence of reported adverse events, serious adverse events, and LVEF < 50%. Secondary endpoints included the change in LVOT-G at rest and during Valsalva maneuvers over the treatment periods, the number of patients demonstrating a hemodynamic response (defined as an LVOT-G < 30 mmHg and < 50 mmHg at rest and during Valsalva, respectively), and the baseline change of LVEF, N-terminal BNP, high sensitivity troponin and NYHA functional class. After 10 weeks of treatment, cohort 1 demonstrated a decreased mean difference in resting LVOT-G of − 29 ± 7.2 mmHg compared to placebo. Cohort 2 demonstrated a mean difference of − 28 ± 7.2 mmHg compared to placebo. After the washout period at 12 weeks, both cohorts had a return to baseline LVOT-G. At 10 weeks, a hemodynamic response with resting LVOT-G < 30 mmHg and a Valsalva gradient of < 50 mmHg was found in 10 out of the 14 cohort one patients and 13 out of the 14 cohort two patients (79% and 93% respectively), whereas it was only 1 out of 12 patients in the placebo group. An observed dose-dependent decrease in LVEF was demonstrated with − 0.6% (standard error 0.084) per mg of aficamten. Two patients receiving aficamten in cohort 2 had reduction in LVEF < 50%; however, on the follow-up echocardiography 2 weeks after the last dose, LVEF returned to baseline for both patients (57% and 71%) with no reported adverse events. Fifty-four percent of patients in cohort 1 reported a change of ≥ 1 NYHA class and 43% of patients in cohort 2 reported the same finding.

A follow-up study in 2023, REDWOOD-HCM Cohort 3, was an open-label study that enlisted patients with symptomatic obstructive HCM that were also on other medications [77]. The study included 13 patients on disopyramide who were given 5–15 mg of aficamten. The results demonstrated a significant reduction in the average resting and post-Valsalva LVOT-G. An improvement in NYHA functional class and cardiac biomarkers was also demonstrated. REDWOOD-HCM Cohort 4 included 41 nHCM patients with NYHA class II/III symptoms and had an established elevated NT-proBNP > 300 pg/ml as well as an LVEF ≥ 60% without LVOT-G whether resting or provoked and no history of reduced LVEF < 45% [78]. All participants received up to three incremental doses of aficamten from 5 to 15 mg daily. The trial lasted over 10 weeks with a 2-week washout period. Findings were consistent with progress in heart failure symptoms and cardiac biomarkers.

The REDWOOD-HCM study was extended into a 5-year trial initially named REDWOOD-HCM OLE (open label extension), and later renamed to FOREST-HCM. This study is an ongoing, open-label extension study that was designed to assess the long-term safety and tolerability of aficamten. The study enrollment included patients with obstructive and nHCM who have completed REDWOOD-HCM. Patients were eligible if they had LVEF ≥ 55% during screening. The patients with oHCM were started on 5 mg of aficamten and doses were adjusted between 5 and 20 mg based on echocardiographic guidance**.** Preliminary data on 45 patients through 1 year was recently presented at the American College of Cardiology meeting [79]. They showed Valsalva LVOT-G < 30 mmHg in 91% of patients and NYHA class improvement ≥ 1 in over 80% of patients. There were no drug discontinuations due to LVEF declines but there was one dose reduction and one interruption due to EF < 50%. Additionally, 78% of the cohort was on a BB and 18% on a CCB.

The SEQUOIA-HCM trial was a phase III, multicenter, randomized, double-blind, placebo controlled trial that was designed with the goal of evaluating the safety and efficacy of aficamten in adults with symptomatic oHCM that have been on background medical therapy for 24 weeks [80]. This was the largest obstructive HCM trial and enrolled 282 adult patients. The primary endpoint was change in pVO_2_ from baseline to 24 weeks assessed through cardiopulmonary exercise testing. There were also ten prespecified secondary outcomes which were hierarchically tested. The secondary outcomes were assessed at 12 and 24 weeks and included changes in the Kansas City Cardiomyopathy Questionnaire-clinical Summary SCORE (KCCQ-CSS), improvement in NYHA functional class ≥ 1, changes in post-Valsalva LVOT-G and the proportion of patients with post-Valsalva LVOT-G < 30 mmHg, and change in the total workload during CPET. Randomization was on a 1:1 basis into aficamten and placebo groups alongside the standard of care treatment. The patients received incremental doses of aficamten in four doses starting at 5 mg daily, guided by echocardiographic parameters. Results from the SEQUOIA-HCM demonstrated that there was a mean change from baseline peak oxygen uptake to week 24 which was 1.8 mL/kg/min (95% CI 1.2–2.3) among patients that received aficamten while it was 0.0 mL/kg/min in the placebo group (95% CI − 0.5 to 0.5) [9]. The placebo-corrected least-squares mean difference was 1.7 mL/kg/min (95% CI 1–2.4) between the groups. A significant benefit to aficamten over placebo was shown in each of the ten secondary endpoints. The aficamten group showed a mean difference in the KCCQ-CSS of 7 points at week 24 (95% CI 5–10) as well as an improvement from baseline in at least one NYHA class in 58.5% of patients, compared to 24.3% of patients in placebo group. The aficamten group also showed a mean difference of − 50 mmHg (95% CI − 57 to − 44) in the post-Valsalva LVOT-G and a post-Valsalva LVOT-G < 30 mmHg in 49.3% of patients when compared to 3.6% in the placebo group. The changes were also evident in patients that were eligible for septal reduction therapies with a mean difference to symptom and hemodynamic changes of 78 days less (95% CI − 100 to − 56) in the aficamten group as compared to placebo. There were eight patients (5.6%) who suffered serious adverse events in the aficamten group compared to 13 patients (9.3%) in the placebo group.

There are several ongoing trials for aficamten. The MAPLE-HCM (Metoprolol vs Aficamten in Patients with LVOT Obstruction on Exercise Capacity in HCM) is a phase III trial testing aficamten compared to metoprolol as first-line therapy in patients with symptomatic oHCM. The primary endpoint is change in pVO_2_ from baseline to 24 weeks measured by CPET. ACACIA-HCM (Assessment Comparing Aficamten to Placebo on Cardiac Endpoints In Adults with Non-Obstructive HCM) is a phase three trial evaluating aficamten in patients with symptomatic nHCM. The primary endpoint is change in KCCQ from baseline to 36 weeks. CEDAR-HCM (Clinical Evaluation of Dosing with Aficamten to Reduce Obstruction in a Pediatric Population in HCM) is a safety and efficacy trial testing aficamten in a pediatric population with symptomatic oHCM. The primary endpoint is change in Valsalva LVOT-G from baseline to week 12.

## Conclusion

Cardiac myosin inhibitors have been proven to be safe and efficacious, as evident from the studies summarized above. Ongoing trials aim to further investigate the outcomes of these patients and to ensure a favorable side effect profile in the long term. At the current juncture, CMIs are considered a second-line treatment for HCM, owing to their relatively recent discovery and cost. In the future, CMIs could potentially replace current standard treatment for HCM and change the trajectory and natural progression of the disease. Further advancements in the topic of HCM are awaited with much anticipation.

## Data Availability

No datasets were generated or analysed during the current study.
